# Three Siblings with Prader-Willi Syndrome: Brief Review of Sleep and Prader-Willi Syndrome

**DOI:** 10.1155/2015/278287

**Published:** 2015-11-03

**Authors:** Arina Bingeliene, Colin M. Shapiro, Sharon A. Chung

**Affiliations:** ^1^Department of Psychiatry, Faculty of Medicine, University of Toronto, Canada; ^2^Toronto Western Hospital, University Health Network, 399 Bathurst Street, 7 Main, Room 422, Toronto, ON, Canada M5T 2S8; ^3^Sleep Research Laboratory, Toronto Western Hospital, 399 Bathurst Street, 7 Main, Room 422, Toronto, ON, Canada M5T 2S8; ^4^Sleep Research Unit, Department of Psychiatry, Toronto Western Hospital, University Health Network, 399 Bathurst Street, 7 Main, Room 422, Toronto, ON, Canada M5T 2S8

## Abstract

Prader-Willi syndrome (PWS) is a genetic disorder characterized by short stature, mental retardation, hypotonia, functionally deficient gonads, and uncontrolled appetite leading to extreme obesity at an early age. Patients with this condition require multidisciplinary medical care, which facilitates a significant improvement in quality of life. PWS is the first human disorder to be attributed to genomic imprinting. Prevalence varies in the literature, ranging from 1 in 8,000 in the Swedish population to 1 in 54,000 in the United Kingdom. Rarely, the genetic mechanism responsible for Prader-Willi syndrome can be inherited. We report a highly unique case of three siblings who share this condition. This report describes a case of two brothers and one half sister with PWS. All three siblings have sleep-related complaints. The sister died at the age of 24 years in her sleep, with the cause of death reported as obstructive sleep apnea. The outcome was positive in both of the brothers' cases as a result of professional medical care and specific tailored recommendations implemented by their mother. A review of the relevant literature vis-à-vis sleep and PWS is provided.

## 1. Brief Review of Sleep and Prader-Willi Syndrome

Prader-Willi Syndrome (PWS) is a disorder caused by the deletion or disruption of genes in the proximal arm of chromosome 15 or by maternal disomy in the proximal arm of chromosome 15. Characteristics commonly associated with this disorder include diminished fetal activity, obesity, hypotonia, mental retardation, short stature, hypogonadotropic hypogonadism, strabismus, and small hands and feet. PWS is the first human disorder to be attributed to genomic imprinting. It results from the loss of imprinted genomic material within the paternal 15q11.2-13 locus. PWS has been reported worldwide, with the majority of cases observed sporadically and occurring without genetic inheritance. Burd et al. have reported a prevalence rate of 1 in 16,062 individuals in the United States [1]. Rarely, the genetic mechanism responsible for Prader-Willi syndrome can be inherited. For example, it is possible for a genetic change that abnormally inactivates genes on the paternal chromosome 15 to be passed from one generation to the next [2].

Sleep disturbances are common among individuals with PWS and may range from central or obstructive sleep apnea (OSA) to narcolepsy [3]. As a result of growth hormone deficiency commonly associated with the condition, current treatments of PWS often involve daily growth hormone therapy. However, the initiation of treatment has been shown to exacerbate existing obstructive sleep apnea, thereby raising serious medical concerns.

In 1999, Richdale et al. [4] conducted a questionnaire study comparing 29 patients with PWS with individuals from an age- and gender-matched control group. Individuals with PWS were found to have sleep disorders with greater frequency than control subjects. Sleep problems included excessive daytime sleepiness (EDS), snoring, and early waking. Notably, sleep problems in PWS were not shown to be associated with body mass index or weight. Excessive daytime sleepiness was a distinctive feature of the group with PWS, and behavioral disturbance in PWS children and adolescents was associated with EDS. EDS appears to be characteristic of PWS and may be related to problems with the sleep-wake cycle and hypothalamic dysfunction.

The diagnostic workup of individuals suspected of having PWS includes genetic testing and a broad endocrinology assessment. If symptoms suggestive of sleep apnea or narcolepsy are endorsed, a sleep study should be performed to confirm the presence of sleep pathology with the addition of multiple sleep latency testing to quantify the degree of excessive daytime sleepiness.

PWS is associated with a broad spectrum of medical and psychiatric disorders, including anxiety disorder (as well as obsessive-compulsive disorder), fragile X syndrome, growth hormone deficiency, hypogonadism, and obesity.

PWS patients require a management approach that is multidisciplinary in nature and that spans a range of issues. Necessary interventions may include initial management of hypotonia or poor feeding, evaluation for hypogonadism or hypopituitarism, management of obesity, monitoring for scoliosis, and therapy for mood, behavioral, and sleep issues [5–8]. Patients with PWS frequently reach adulthood and are able to function in a group home setting, performing vocational work or attending community college classes. However, complications from hypogonadism (e.g., osteoporosis/pathologic fracture) and morbid obesity (e.g., type 2 diabetes mellitus, cor pulmonale) may shorten the life expectancy of these individuals and poor mental health or behavioral issues (e.g., temper tantrums, stubbornness) may adversely affect quality of life.

We present here a case report series of two brothers (Patients A and B) and a half sister (Patient C) with genetically confirmed diagnosis of PWS.

Patient A and Patient B are half brothers to Patient C. They live with their mother, sharing a single bedroom with separate beds. The brothers were born in Kenya and are of Indian descent. The father is not involved in the boys' lives and their parents are divorced. Both brothers have PWS.

In terms of family history, the brothers' maternal grandmother and two aunts have a history of mood disorder, controlled with medications, and they have never been hospitalized. The brothers' paternal grandfather has a history of PWS and mental health problems.

Patient A and Patient B met the following diagnostic criteria (eight total points are required for a diagnosis of PWS at the age of 3 years to adulthood, including at least five from the major criteria list [9]).

Major criteria are as follows (1 point for each of the following features): neonatal and infantile central hypotonia with poor ability to suckle; feeding problems in infancy; central obesity; dysmorphic face features narrow face and almond shape eyes (Patient B more prominent than Patient A); hypogonadism; global developmental delay and mental retardation, learning problems requiring individual educational plan; excessive appetite and obsession with food (their mother had to put the lock on the fridge door and the kitchen door); deletion 15q11–13. Minor criteria are as follows (1/2 point for each of the following features): speech articulation defects; sleep disturbance; other features will be mentioned individually for Patient A and Patient B in the following cases description.

## 2. Case Report A

Patient A is a 20-year-old man with a history of PWS. His diagnosis was confirmed by genetic testing when he was 11 years old. He has a paternal deletion like both his brother and half sister. As part of this syndrome he was diagnosed with hypogonadotropic hypogonadism, growth hormone deficiency, and low coagulation factor FVII. Patient A receives testosterone injections on a monthly basis and receives growth hormone injection 6 days per week. He has been prescribed Modafinil (to control his appetite) and also takes vitamin D, vitamin B6, calcium, and multivitamin complex supplements. He is on the “red-yellow-green” diet [10] and has achieved good success with this.

Patient A has a mild deficiency of coagulation factor FVII, which has been deemed as being unlikely to cause him any bleeding symptomatology. He has no family history of any bleeding disorder and no known history of family members with low FVII. He is seen regularly in follow-up in the Bleeding Disorders Clinic at The Hospital for Sick Children (Toronto). He has been advised not to take aspirin and to follow a few precautions to prevent possible excessive bleeding. These include wearing a helmet for any activity that could potentially be associated with head trauma and receiving replacement therapy in the case of major surgeries.

He has no known drug allergies and his immunizations are up to date. He has no history of psychiatric illness and does not smoke or take recreational drugs. He graduated from Grade 12 when he was 18 years old. He was enrolled in a special education plan and his academic performance was good. He likes mathematics.

His physical examination showed the following pertinent features. He was 166 cm tall and his weight was 80.5 kg. His body mass index (BMI) was 29.2 kg per m^2^. He demonstrated mild hypotonia and hyporeflexia at a grade of 1+/1+, and his plantar responses were flexor. He possessed a full range of neck movement, but on anterior flexion his fingertips were approximately 20 cm off his toes. He did not have clear dysmorphic features. His gait was normal and Romberg's test was negative. His cranial nerves were normal. He was somewhat argumentative (the mother reported this as “typical of being a teenager”) and he was found to sometimes “talk back” to his mother.

### 2.1. Sleep Assessment

An overnight polysomnographic (PSG) sleep assessment was conducted to rule out possible OSA. This sleep study was performed at the Youthdale Child and Adolescent Sleep Centre when Patient A was 18 years old. The study found that he had normal sleep onset latency and sleep efficiency. He demonstrated moderate rapid eye movement- (REM-) related OSA (a moderately increased apnea/hypopnea index (AHI) of 21 events per hour), but his AHI over his total sleep time was normal (AHI was 3.9 per hour). No significant oxygen desaturation or snoring was recorded. Sleep fragmentation was clearly observed across the night, with Patient A experiencing a significantly increased number of intervening awakenings.

Following this first study, it was explained to Patient A that PWS is associated with a higher frequency of OSA and that a correlation between poor sleep (including sleep fragmentation) and increased body weight has been observed. It was recommended that he continue with his diet and that he engage in exercise in order to improve his weight control. He was advised to repeat the sleep study in two years' time to reassess the severity of his OSA.

A second PSG study was conducted two and a half years later when he was 21 years old to reassess his OSA. At this time, he described feeling alert during the day and reported his mood as “happy.” He was performing a volunteer job.

The PSG showed an improvement in his sleep efficiency, and his AHI was normal both over his total sleep time and in REM sleep. The number of arousal times observed during the study decreased in comparison to the previous assessment, with both features found to be in the normal range. However the sleep study showed a decreased amount of slow wave sleep (SWS) during the night. At this point, his BMI remained steady at 29 kg per m^2^. Strategies to enhance SWS (including increasing aerobic exercise and raising body temperature before going to bed by, e.g., taking a hot bath) were discussed.

## 3. Case Report B

Patient B, brother to Patient A and half brother to Patient C, is a 23-year-old man who was referred to the sleep clinic for the first time when he was 18 years old to rule out OSA and to assess his sleep-related problems. He and his mother were concerned about his sleep given that he reported feeling tired most of the day. During sleep he was observed to snore loudly, experience apneic-like events during which he stopped breathing, talk in his sleep, and occasionally experience jerky body movements.

Patient B is prone to frequent upper-airway infections. The PWS genetic test was performed when he was 14 years old and confirmed the diagnosis as a paternal chromosome deletion.

Patient B was delivered by cesarean section (due to a failure of labor to progress). His birth weight was 5 lbs. His early childhood developmental milestones were delayed and he experienced feeding difficulties. He is on risperidone 1.5 mg daily and testosterone 0.25 mg daily, and he was on human growth hormone injections previously. He had not been prescribed Modafinil as the main treatment goals for him were to improve his nighttime sleep quality in order to reduce his daytime fatigue. He is also adhering to the “red-yellow-green” diet for PWS patients [10] and has achieved good body weight control success.

Patient B has cryptorchidism and a history of thyroid hypofunction, which is controlled with medications, and osteopenia due to androgen deficiency.

He also has a history of bipolar disorder, type I, for which he is taking valproic acid, quetiapine, Topiramate, and lorazepam. He had previously been hospitalized for his mood disorder with manic episode exacerbation when he was 21 years old.

He graduated from Grade 12 when he was 18 years old. He was enrolled in a special education plan and his academic performance was good. He currently works as a retail clerical assistant. He has learning difficulty and is exempt from Education Quality and Accountability Office (EQAO) tests, both for mathematics in Grade 9 and for the literacy test in Grade 10.

His first physical examination in our sleep clinic was performed when he was 21 years old and it showed the following pertinent features. He was 159 cm tall and his weight was 73.4 kg. His BMI was 29 kg per m^2^. He demonstrated decreased muscle tone without hyperflexibility. A neurological exam showed a slowed response and evinced echolalic speech. Cranial nerves II to XII were normal. His deep tendon reflexes were hypoactive 1+/1+, his plantar responses were flexor, his gait was normal, Romberg's test was negative, and his sensory examination was grossly intact.

### 3.1. Sleep Assessment

An initial PSG study was conducted when Patient B was 18 years old to investigate possible OSA and parasomnia. A multiple sleep latency test (MSLT) was carried out on the following day to assess the severity and nature of his excessive daytime sleepiness. The sleep study showed normal sleep onset latency and sleep efficiency, mildly increased AHI in total sleep (12.8/h), and moderately increased AHI in REM sleep (20.4/h). The increase during REM was predominantly due to central respiratory events associated with significant oxygen desaturations (minimum of 74%) and occasional mild to loud snoring. The test also showed that his sleep was fragmented, with an arousal index of 12.2 per hour. There were some polysomnographic (PSG) features suggestive of NREM parasomnia.

The mean sleep latency observed on the MSLT was normal. Given that central apnea episodes are occasionally associated with brain tumors and that the most common cause of OSA in the pediatric population is tonsillar hypertrophy, Patient B was referred for neurological and ENT assessments to rule out the presence of other medical disorders.

A second PSG study was conducted three years later when Patient B was 21 years old. He was then observed to have difficulties initiating sleep, moderate central sleep apnea syndrome with an AHI of 19.4 per hour in total sleep, and a central apnea index of 18.2 per hour. Bilevel positive airway pressure (BiPAP) treatment was initiated. During the BiPAP titration, Patient B was found to require an unexpectedly high pressure (17/12 cm H_2_O) potentially due to the hypotonia typically associated with PWS. Patient B's subjective daytime alertness and his mood improved after this treatment with BiPAP was initiated.

### 3.2. Computed Tomography

Computed tomography (CT) of the head was conducted when Patient B was 22 years old. It showed nonspecific frontal subcortical white matter changes. No mass was identified. Following neuroimaging, Patient B was seen by a neurologist and he and his mother were informed that individuals with PWS have been found in the literature to demonstrate patchy white matter changes of unknown pathogenesis. These lesions may be related to underlying metabolic disorders experienced by these individuals, which predispose these individuals to premature cardiovascular disease in the microvasculature. A magnetic resonance imaging scan was not recommended at this time.

### 3.3. Sleep Assessment Follow-Up

A follow-up PSG study was conducted for Patient B when he was 23 years old. At this time his BMI remained at 29 kg per m^2^. The study demonstrated successful breathing control with BiPAP at 15/10 cm of water pressure. Patient B was found to have normal sleep onset latency and sleep efficiency and decreased SWS. His medications at the time of assessment were lithium 900 mg, levothyroxine 0.05 mg, Topiramate 225 mg, calcium 1200 mg, omega 3, vitamin D, and a multivitamin complex.

## 4. Case Report C

This individual, Patient C, was a half sister to the other patients assessed as part of this report. She was not seen by the authors. The complete medical file for Patient C was not available for our review; therefore we could not provide the full description of clinical features of PWS for this patient. Patient C's mother who currently resides in London, UK, was contacted and asked to provide details regarding her daughter's medical condition. Patient C shared a father with Patients A and B and was a child of his first marriage. She had a history of PWS, cerebral palsy, and behavioral and sleep-related problems. Genetic analysis had confirmed her PWS diagnosis as paternal deletion. She was 24 years of age at the time of her death and was obese. Her death certificate reports her cause of death as obstructive sleep apnea.

During a posthumous assessment conducted over the telephone, Patient C's mother confirmed that she believes her daughter had obstructive sleep apnea. Based on the STOP-BANG questionnaire which screens for risk factors related to apnea [11], the history provided by the mother indicates that Patient C would have had a 70% chance of having sleep apnea. Patient C's mother expressed surprise that an overnight PSG assessment was never conducted for her daughter.

In addition to suspected OSA, Patient C had cerebral palsy and was using a wheelchair. She had been prescribed sedating medications for her behavior-related problems.

## 5. Discussion

The case reports detailed here provide a unique example of inherited PWS observed in three siblings. This report confirms that not all PWS patients necessarily display all known sleep- or mood-related symptoms of the disorder. Further, it indicates that elevated BMI is not always accompanied by sleep apnea.

The trajectory of the two male patients provides an excellent example of how multidisciplinary approaches to PWS can achieve success. In combination with a dedicated support network in the form of the patients' mother who provided significant support in terms of following recommendations made by medical professionals, the interventions detailed above helped to significantly improve the quality of life of Patients B and C as they lived with this untreatable genetic condition.

While central sleep apnea is thought to be more prevalent in children, the cases presented above demonstrate the need for continued monitoring into adulthood. Sleep architectural changes were primarily investigated in Patients B and C as part of the diagnostic procedure for sleep-related breathing disorder. However, this report demonstrates that alterations in sleep architecture (including reduced SWS and increased arousal index) may be indicative of insomnia or may occur as a side effect of Modafinil, requiring treatment. Similarly, this suggests the necessity of follow-up throughout adulthood to ensure appropriate and timely treatment of symptoms.
